# Impact of skin flap elevation technique on the extent of gustatory sweating after superficial parotidectomy: a comparative study

**DOI:** 10.1186/s12893-026-03614-8

**Published:** 2026-02-20

**Authors:** Mesut Karataş, Osman Durgut, Fevzi Solmaz, Oğuzhan Dikici, Sündüz Gençay, Hakkı Caner İnan, Basri Cangül

**Affiliations:** Department of Otorhinolaryngology–Head and Neck Surgery, University of Health Sciences, Bursa Yüksek İhtisas Training and Research Hospital, Bursa, Türkiye

**Keywords:** Frey syndrome, Superficial parotidectomy, Skin flap elevation, Starch–iodine test, Monopolar cautery, Cold scalpel

## Abstract

**Background:**

Frey syndrome is a frequent long-term complication following superficial parotidectomy, with reported prevalence varying widely according to diagnostic methods and follow-up duration. Although most studies focus on the presence of Frey syndrome, limited data are available regarding the influence of surgical technique on the *extent* or *severity* of gustatory sweating.

**Methods:**

This single-center, prospective observational study included adult patients who underwent superficial parotidectomy for benign parotid gland lesions between March 2021 and March 2023. Frey syndrome was evaluated using the starch–iodine test performed at a median of 12 months postoperatively. Test positivity, clinical symptoms, and the area of starch–iodine test positivity (cm²) were recorded. Patients were grouped according to skin flap elevation technique (cold scalpel vs. monopolar cautery). Tumor size and postoperative outcomes were analyzed, and effect size estimates were calculated to complement statistical significance testing.

**Results:**

Thirty patients were included (median age: 59 years; 70% male). The starch–iodine test was positive in 13 patients (43.3%), whereas clinical Frey syndrome was observed in only 2 patients (6.7%). Test positivity rates did not differ significantly between the cold scalpel and monopolar cautery groups (35.7% vs. 50.0%, *p* = 0.484). Among patients with a positive test, the monopolar cautery group demonstrated a larger median area of gustatory sweating compared with the cold scalpel group (15.85 vs. 8.00 cm²), although this difference did not reach statistical significance (*p* = 0.284); a moderate effect size was observed.

**Conclusion:**

While the incidence of Frey syndrome was similar between surgical techniques, monopolar cautery was associated with a trend toward a greater extent of gustatory sweating. Among patients with a positive starch–iodine test, differences were observed in the extent of gustatory sweating according to the skin flap elevation technique; however, these findings should be interpreted descriptively and do not imply a causal effect on disease severity. These findings suggest that the skin flap elevation technique may influence the extent of subclinical gustatory sweating, rather than the clinical incidence or severity of Frey syndrome.

## Introduction

Parotid gland surgery, particularly superficial parotidectomy, is widely performed for the management of benign parotid tumors and is generally associated with favorable oncological and functional outcomes. Nevertheless, Frey syndrome remains one of the most common long-term complications following parotidectomy, characterized by gustatory sweating and flushing in the preauricular and temporal regions. The reported prevalence of Frey syndrome varies considerably in the literature, largely depending on diagnostic methods, follow-up duration, and whether objective testing is routinely employed [1–3].

Frey syndrome is thought to result from aberrant regeneration of parasympathetic fibers of the auriculotemporal nerve, which, after surgical injury, reinnervate the sweat glands of the overlying skin. While many patients remain asymptomatic, objective diagnostic methods such as the starch–iodine test positivity test frequently reveal subclinical gustatory sweating, indicating that the true prevalence of Frey syndrome is substantially higher than that suggested by patient-reported symptoms alone [1–3]. This discrepancy underscores the importance of incorporating objective assessment tools when evaluating postoperative outcomes following parotidectomy.

Numerous surgical strategies have been proposed to reduce the incidence of Frey syndrome, including interpositional barrier techniques, muscle or fascial flaps, and modifications of the surgical approach. Systematic reviews and meta-analyses have demonstrated that these preventive techniques may reduce the occurrence of Frey syndrome; however, none has been universally adopted as standard practice, and their effectiveness varies across studies [4, 5]. In addition, patient- and tumor-related factors, such as tumor size and extent of resection, have been investigated as potential risk factors, with inconsistent results reported in the literature [6, 7].

Beyond preventive measures, the role of surgical technique during skin flap elevation has received comparatively little attention. Energy-based dissection methods, including monopolar cautery and harmonic scalpel devices, are widely used in contemporary parotid surgery due to their efficiency and hemostatic advantages. Unlike ultrasonic or radiofrequency devices, monopolar electrocautery relies on thermal energy with a wider and less controlled lateral thermal spread, which may result in greater collateral tissue and neural injury. Consequently, these techniques may produce varying degrees of thermal spread and collateral tissue injury compared with cold scalpel dissection, potentially influencing neural damage and subsequent aberrant reinnervation [8, 9]. While previous studies have primarily focused on the presence or absence of Frey syndrome, data regarding the impact of surgical technique on the *extent* or *severity* of gustatory sweating remain limited.

Therefore, the aim of the present study was to evaluate Frey syndrome following superficial parotidectomy using objective starch–iodine testing, with a particular focus on comparing cold scalpel and monopolar cautery techniques during skin flap elevation. In addition to assessing test positivity and clinical symptoms, we sought to quantify the extent of gustatory sweating and to explore its relationship with tumor size, thereby providing descriptive insight into factors associated with differences in the extent of Frey syndrome among affected patients.

## Materials and methods

### Study design and ethical approval

This study was conducted as a single-center, prospective, observational study at Bursa Yüksek İhtisas Training and Research Hospital. The study protocol was reviewed and approved by the Clinical Research Ethics Committee of Bursa Yüksek İhtisas Training and Research Hospital (protocol code: 2011-KAEK-25 / 2021-03-08). The study was performed in accordance with the principles of the Declaration of Helsinki.

### Study population

Patients aged 18 years or older who underwent superficial parotidectomy for benign parotid gland lesions at our institution were evaluated for inclusion. Patients with malignant pathology, a history of previous parotid surgery, revision procedures, or incomplete postoperative follow-up were excluded from the study.

### Surgical technique

All superficial parotidectomies were performed using a standard modified Blair incision, which is routinely employed in our institution. All patients underwent superficial parotidectomy using a standard surgical approach. During skin flap elevation, either a cold scalpel or monopolar cautery technique was used. Monopolar flap elevation was performed using a conventional electrosurgical generator in standard cutting–coagulation mode with a microdissection needle tip; no advanced tissue-feedback or dynamic tissue response technologies were used. Surgical technique selection (cold scalpel versus monopolar cautery) was based on surgeon preference and routine clinical practice rather than random allocation. Both techniques are routinely and safely applied in our clinic, and the choice of technique depended solely on the routine preference of the operating surgeon. The study was not randomized, and patients were grouped according to the technique used during surgery. Variables such as flap thickness, SMAS handling, interpositional barrier use, and the extent of nerve dissection were not systematically measured or standardized and were therefore not included as stratification or adjustment variables in the analysis.

No additional surgical intervention beyond standard care was performed for the purposes of the study.

### Assessment of Frey Syndrome

Patients were routinely followed postoperatively in the outpatient clinic. Frey syndrome was evaluated using the starch–iodine test, which is a non-invasive and widely accepted diagnostic method for detecting gustatory sweating.

Because symptoms of Frey syndrome may develop several months after surgery, the starch–iodine test was performed at a median of 12 months postoperatively. After an overnight fast, the parotid region was painted with 10% starch–iodine test positivity solution and allowed to dry, followed by application of starch powder. Patients were then stimulated gustatorily, and the surgical area was assessed at 0, 30, and 60 min. The appearance of blue–black discoloration in the parotidectomy region was considered indicative of a positive test result. Both sides of the face were evaluated to provide an internal control.

In patients with a starch–iodine test positivity, the area of discoloration was measured and recorded in square centimeters (cm²). The starch–iodine positive area was delineated based on visible blue–black discoloration after gustatory stimulation and measured manually in square centimeters by a single experienced clinician during postoperative follow-up; assessor blinding and formal inter- or intra-observer reliability testing were not performed.

Clinical Frey syndrome was defined as the presence of patient-reported symptoms of gustatory sweating during follow-up.

### Outcome measures

The primary outcome of the study was defined as the extent of gustatory sweating, quantified by the area of starch–iodine positivity (cm²) among test-positive patients, whereas starch–iodine test positivity itself was analyzed as a secondary outcome. Quantitative analysis of the starch–iodine positive area was performed exclusively among patients with starch–iodine test positivity result. This subset analysis was designed as a descriptive comparison of the extent of gustatory sweating in affected individuals, rather than a population-level risk assessment. Additional secondary outcomes included the presence of clinical Frey syndrome, tumor size, and postoperative transient facial nerve dysfunction.

### Statistical analysis

Statistical analyses were performed using IBM SPSS Statistics for Windows, version 27.0 (IBM Corp., Armonk, NY, USA). Normality of continuous variables was assessed using the Shapiro–Wilk test. which demonstrated non-normal distribution for most variables.

Continuous variables were expressed as median and interquartile range (IQR), while categorical variables were presented as counts and percentages. Comparisons between groups were performed using the Mann–Whitney U test for continuous variables and the Chi-square test or Fisher’s exact test for categorical variables, as appropriate. Correlations between tumor size and starch–iodine test positivity area were assessed using Spearman’s rank correlation coefficient.

Effect size for the Mann–Whitney U test was calculated using the formula *r = Z / √N*. Odds ratios were not calculated in comparisons involving zero cell counts. A two-sided p value < 0.05 was considered statistically significant.

## Results

A total of 30 patients who underwent superficial parotidectomy were included in the final analysis. The median age of the study population was 59 years (IQR: 54–63.5), and 70% of the patients were male. Sixteen patients (53.3%) underwent skin flap elevation using monopolar cautery, while fourteen patients (46.7%) were treated using a cold scalpel technique. Analyses involving tumor size included 29 patients due to missing tumor size data in one case.

Baseline demographic, clinical, surgical, and pathological characteristics of the study population are summarized in Table 1. No clinically relevant differences were observed between the two surgical technique groups with respect to baseline characteristics. The proportion of female patients was similar between the cold scalpel and monopolar cautery groups (28.6% vs. 31.3%, Fisher’s exact test *p* = 1.000).

**Table 1:** Baseline demographic, clinical, surgical, and pathological characteristics of the study population according to skin flap elevation technique

Variable	Total (*n* = 30)	Cold scalpel (*n* = 14)	Monopolar cautery (*n* = 16)	*p* value
Demographic characteristics				
Age, years, median (IQR)	59 (54–63.5)	59 (54–63.5)	59 (55–64)	0.842†
Male sex, n (%)	21 (70.0)	10 (71.4)	11 (68.8)	1.000‡
Female sex, n (%)	9 (30.0)	4 (28.6)	5 (31.3)	1.000‡
Lifestyle and comorbidities				
Smoking, n (%)	11 (36.7)	5 (35.7)	6 (37.5)	1.000‡
Diabetes mellitus, n (%)	3 (10.0)	1 (7.1)	2 (12.5)	1.000‡
Hypertension, n (%)	0 (0)	0 (0)	0 (0)	—
Benign prostatic hyperplasia, n (%)	2 (6.7)	1 (7.1)	1 (6.3)	1.000‡
Coronary artery disease, n (%)	2 (6.7)	1 (7.1)	1 (6.3)	1.000‡
Final histopathological diagnosis				
Pleomorphic adenoma, n (%)	15 (50.0)	—	—	—
Warthin tumor, n (%)	8 (26.7)	—	—	—
Epithelial neoplasm, n (%)	3 (10.0)	—	—	—
Other benign tumors, n (%)	4 (13.3)			
Tumor and specimen characteristics				
Tumor size, cm, median (IQR)	8.1 (4.0–13.5)	8.1 (4.0–13.5)	8.1 (4.0–13.8)	0.842†
Specimen size, cm, median (IQR)	31.2 (24.1–46.8)	31.2 (24.1–46.8)	31.2 (24.1–53.4)	0.611†
Surgical characteristics				
Surgical technique, n (%)	—	14 (46.7)	16 (53.3)	—
Surgical site – Region 1, n (%)	17 (56.7)	8 (57.1)	9 (56.3)	1.000‡
Surgical site – Region 2, n (%)	13 (43.3)	6 (42.9)	7 (43.8)	1.000‡

Data are presented as median (interquartile range [IQR]) for continuous variables and as frequency (percentage) for categorical variables. Other benign tumors include less frequent benign histopathological subtypes. There were no statistically significant differences between the cold scalpel and monopolar cautery groups regarding baseline characteristics (*p* > 0.05)

Clinical Frey syndrome was observed in two patients (6.7%), both of whom were in the monopolar cautery group. However, the difference between the cold scalpel and monopolar cautery techniques was not statistically significant (*p* = 0.485). The starch–iodine test was positive in 13 patients (43.3%). Test positivity rates did not differ significantly between the cold scalpel and monopolar cautery groups (35.7% vs. 50.0%, respectively; *p* = 0.484, Table 2).

**Table 2:** Comparison of surgical outcomes between cold scalpel and monopolar cautery techniques

Variable	Cold scalpel (*n* = 14)	Monopolar cautery (*n* = 16)	*p* value
Frey syndrome, n (%)	0 (0)	2 (12.5)	0.485†
Starch–iodine test positivity, n (%)	5 (35.7)	8 (50.0)	0.484†
Tumor size, cm, median (IQR)	8.1 (4.0–13.5)	8.1 (4.0–13.8)	0.842‡
Specimen size, cm, median (IQR)	31.2 (24.1–46.8)	31.2 (24.1–53.4)	0.611‡
Transient facial paralysis, n (%)	0 (0)	1 (6.3)	1.000†

Data are presented as median (interquartile range) or n (%). † Fisher’s exact test. ‡ Mann–Whitney U test

Tumor size did not differ significantly between patients with positive and negative starch–iodine test results. The comparison of tumor size according to starch–iodine test status is presented in Table 3.

**Table 3:** Tumor size according to starch–iodine test results

Variable	Test Negative (*n* = 16)	Test Positive (*n* = 13)	*p* value
Tumor size, cm, median (IQR)	6.45 (3.25–7.73)	8.10 (3.85–13.75)	0.329†

Data are presented as median (interquartile range). † Mann–Whitney U test (exact significance). There was no statistically significant difference in tumor size between patients with negative and positive starch–iodine test results (*p* = 0.329). This finding suggests that, within this study cohort, starch–iodine test positivity (subclinical Frey syndrome) was not primarily driven by initial tumor size. One patient was excluded from this analysis due to missing tumor size data

Among patients with a starch–iodine test positivity, the size of the starch–iodine test positive area differed between surgical techniques. The difference in starch–iodine test positivity area between the cold scalpel and monopolar cautery groups was not statistically significant (*p* = 0.284), with a small effect size (*r* = 0.14, 95% CI: −0.23 to 0.48). The monopolar cautery group demonstrated a larger median positive test area compared with the cold scalpel group (15.85 [4.63–64.50] cm² vs. 8.00 [3.75–9.50] cm², respectively), although this difference did not reach statistical significance (*p* = 0.284, Table 4).

**Table 4:** Comparison of starch–iodine test positivity area (cm²) according to surgical technique

Variable	Cold scalpel (*n* = 5)	Monopolar cautery (*n* = 8)	*p* value
Starch–iodine test positivity area, cm**²**	8.0 (3.75–9.50)	15.85 (4.63–64.50)	0.284†
Effect size (r): 0.14 95% confidence interval: −0.23 to 0.48			

Although the difference in the starch–iodine test positive area did not reach statistical significance (*p* = 0.284), effect size (*r* ≈ 0.14) was observed, suggesting a trend toward a larger extent of gustatory sweating in the monopolar cautery group

Among patients with starch–iodine test positivity, a moderate positive correlation was observed between tumor size and the extent of starch–iodine test positivity (Spearman’s ρ = 0.425, 95% CI: −0.16 to 0.79); however, this association did not reach statistical significance (*p* = 0.321; Table 5).

**Table 5:** Correlation between tumor size and starch–iodine test positivity area

Variables	Spearman’s ρ	95% Cl	*p* value	
Tumor size (cm) vs. starch–iodine test positivity area (cm²)	0.425	−0.16 to 0.79	0.321	

Spearman’s rank correlation analysis (*n* = 13), including only patients with starch–iodine test positivity result. Confidence intervals were calculated using Fisher’s z transformation. A moderate positive correlation (ρ = 0.425) was observed between tumor size and starch–iodine test positivity area; however, this association did not reach statistical significance (*p* = 0.321), likely due to the limited sample size of this exploratory analysis

## Discussion

Frey syndrome remains one of the most frequent and bothersome complications following superficial parotidectomy, with reported prevalence rates ranging widely depending on diagnostic methods and follow-up duration [1–3]. Previous studies have reported wide variability in the prevalence of Frey syndrome after parotidectomy, largely depending on the diagnostic method used and the duration of follow-up, with objectively detected subclinical gustatory sweating being substantially more common than clinically apparent symptoms [10]. While most previous studies have focused on the presence or absence of Frey syndrome, fewer have attempted to quantify its extent, and none have directly compared skin flap elevation techniques as a potential determinant of disease severity. In this context, the present study provides novel by evaluating not only starch–iodine test positivity but also the extent of gustatory sweating in relation to surgical technique.

In our cohort, the overall rate of starch–iodine test positivity was 43.3%, whereas clinically symptomatic Frey syndrome was observed in only 6.7% of patients. This discrepancy highlights the well-recognized distinction between subclinical and clinical Frey syndrome and underscores the importance of objective testing methods. The observed rates are consistent with previous reports demonstrating that subclinical gustatory sweating is substantially more common than patient-reported symptoms [2, 3].

When comparing surgical techniques, neither the incidence of clinical Frey syndrome nor starch–iodine test positivity differed significantly between the cold scalpel and monopolar cautery groups. However, among patients with a starch–iodine test positivity, the monopolar cautery technique was associated with a larger median area of gustatory sweating. Although this difference did not reach statistical significance, the presence of a moderate effect size suggests a potentially meaningful clinical signal. Importantly, effect size estimation is increasingly emphasized in contemporary surgical literature, particularly in exploratory studies with limited sample sizes, as it provides information beyond p values alone. Effect size provides an estimate of the magnitude of the observed difference between groups and offers complementary information to p values, particularly in exploratory studies with limited sample sizes.

The observed trend toward a larger affected area in the monopolar cautery group may be explained by differences in tissue injury patterns during flap elevation. Previous studies evaluating different flap elevation and dissection techniques have discussed potential associations between tissue handling, thermal profiles, and postoperative neural regeneration; however, direct evidence linking these factors to the extent of gustatory sweating remains limited [11]. Monopolar cautery has been associated with greater thermal spread in experimental and surgical settings; however, its potential impact on parasympathetic fibers of the auriculotemporal nerve remains speculative and was not directly assessed in the present study [5, 9]. This broader zone of injury could theoretically facilitate aberrant reinnervation of sweat glands during the healing process, leading to a wider distribution of gustatory sweating. In contrast, cold scalpel dissection is more likely to preserve tissue planes and limit collateral neural damage. This hypothesis is biologically plausible, as thermal nerve injury is known to promote aberrant regeneration of parasympathetic fibers toward cutaneous sweat glands, a mechanism that is central to the development of Frey syndrome. While this hypothesis remains speculative, it is biologically plausible and consistent with established concepts regarding the pathophysiology of Frey syndrome [12]. Any discussion regarding potential thermal effects should be interpreted as speculative and hypothesis-generating, as this study was not designed to investigate underlying mechanisms.

Tumor size did not differ significantly between patients with positive and negative starch–iodine test results, suggesting that the occurrence of subclinical Frey syndrome in this cohort was not primarily driven by lesion size alone. Nevertheless, a moderate positive correlation was observed between tumor size and the extent of starch–iodine test positivity among affected patients. Although this association did not reach statistical significance, likely due to the small number of positive cases, it raises the possibility that larger resections may predispose to a greater area of aberrant sweating once Frey syndrome develops [7].

Our findings are consistent with reports indicating that surgical technique may not significantly alter the incidence of Frey syndrome, while potentially influencing its anatomical extent when objectively assessed [13]. From a clinical perspective, the findings highlight descriptive differences in the extent of Frey syndrome observed across surgical techniques among affected patients, without implying a causal effect. Because area measurements were restricted to starch–iodine test–positive patients, this comparison represents a selection-conditioned analysis and does not permit causal inference regarding the effect of surgical technique on the development of Frey syndrome. Even in the absence of statistically significant differences in incidence, an increased extent of gustatory sweating may have important implications for patient comfort and quality of life. Surgeons may therefore consider the potential long-term functional consequences of flap elevation techniques, particularly in patients at higher risk for Frey syndrome [4, 5]. Although differences in the extent of starch–iodine test positivity were observed between surgical techniques, these differences did not reach statistical significance and should be interpreted descriptively within the limitations of the study design.

Several limitations should be acknowledged. The study was non-randomized and observational and the choice of surgical technique depended on surgeon preference, introducing potential selection bias. In addition, the non-randomized design and surgeon-dependent selection of surgical technique may introduce residual confounding and potential clustering by surgeon, which could not be formally accounted for in this exploratory analysis. Moreover, the absence of stratification for known surgical modifiers of Frey syndrome risk—including surgeon-related factors, flap thickness, SMAS handling, interpositional barrier use, and the extent of nerve dissection—represents an important limitation. These factors should be systematically addressed in future studies employing standardized surgical protocols. Furthermore, the measurement of the starch–iodine positive area was performed by a single, non-blinded assessor using a manual delineation method, and inter- or intra-observer reliability was not assessed. This may introduce measurement-related variability and limits the precision of the quantitative endpoint. Despite the inclusion of 95% confidence intervals for effect size and correlation estimates, the limited sample size, especially in analyses restricted to starch–iodine test–positive patients, restricts the precision and generalizability of these findings. The sample size was relatively small, limiting statistical power, particularly for subgroup and correlation analyses. Additionally, quality-of-life measures specific to Frey syndrome were not included. Future randomized studies employing standardized surgical techniques and uniform objective and patient-reported assessment protocols are necessary to confirm the observed trends and clarify the impact of flap elevation technique on both the incidence and extent of Frey syndrome. Despite these limitations, the study provides hypothesis-generating data that may inform future research.

In conclusion, while the incidence of Frey syndrome did not differ significantly between cold scalpel and monopolar cautery techniques, monopolar cautery was associated with a trend toward a larger extent of gustatory sweating. Among patients with starch–iodine test positivity, differences were descriptively observed in the extent of Frey syndrome according to the method of skin flap elevation; however, these findings do not imply a causal effect on disease severity. Accordingly, these results should be interpreted as exploratory and hypothesis-generating, describing observed differences in the extent of gustatory sweating among affected patients rather than causal effects of surgical technique. Larger, prospective, and ideally randomized studies incorporating both objective measurements and patient-reported outcomes are warranted to further explore these observations.

## Data Availability

The datasets generated and/or analyzed during the current study are available from the corresponding author upon reasonable request.
